# Gallbladder fossa volume decreased in livers without gallbladders: A cadaveric study

**DOI:** 10.1371/journal.pone.0257848

**Published:** 2021-09-23

**Authors:** Diana C. J. Rhodes, Ronald F. Walser, Jessica A. Rhodes

**Affiliations:** 1 Department of Anatomy, Pacific Northwest University of Health Sciences, Yakima, Washington, United States of America; 2 Department of Genetics, Stanford University, Palo Alto, California, United States of America; University of Alberta, CANADA

## Abstract

The gallbladder normally lies within a fossa on the visceral surface of the liver. The primary purpose of this study was to determine whether the volume of this fossa was reduced after cholecystectomy. Livers were obtained from embalmed cadavers of 19 females and 15 males with a mean age of 84.1 ± 10.8 yrs. The presence of a gallbladder was assessed, the volume of the irregularly-shaped gallbladder fossa determined from a mold of the fossa, and the dimensions of each fossa were estimated. The mean volume of gallbladder fossae from livers with gallbladders (n = 26; 13 females and 13 males) was 31.01 ± 17.82 ml, which was significantly greater than fossae in livers without gallbladders (n = 8, 6 females, 2 males) which was 8.75 ± 4.72 ml (*P*<0.0001). This difference still was significant after correcting fossa volume for overall liver weight and length of the femur. Livers with gallbladders had significantly larger dimensions (depth, length, and width) of their fossae molds than did livers without gallbladders (*P*<0.05). The largest percentage difference between the two groups in these dimensions was in the fossae depth, and there was a significant, positive correlation between all three of these dimensions and the overall volume of the fossae. Even looking only at female livers which tend to be smaller, gallbladder fossa volume was reduced in livers without a gallbladder. Thus, the present study demonstrated that the mean gallbladder fossa volume was significantly decreased in livers lacking gallbladders, even after correcting for the liver weight and size of the individual. While the mechanisms behind these changes in fossa volume currently are unknown, alterations in mechanical pressure relayed to adjacent liver cells after gallbladder removal may play a role in these fossa volume differences.

## Introduction

The gallbladder, which stores bile produced by the liver, normally is located in the gallbladder fossa, a depression on the visceral surface of the liver located between the right and quadrate anatomical liver lobes [1]. Surgical removal of the gallbladder, cholecystectomy, is one of the most commonly performed surgeries, typically being performed in individuals with symptomatic gallstones [2]. Normally, the liver maintains a stable weight due to a very slow hepatocyte proliferative rate [3]. However, unlike many other tissues, the liver has a remarkable regenerative capacity after damage from disease or surgical removal, with the liver often returning back to 100% of its original size [3]. Imaging studies that examined the gallbladder fossa in patients a few days after cholecystectomy found mild edema or free fluid in the fossa [4, 5]. In the present study, we hoped to begin to establish a normal range for the volume of this fossa in individuals with a gallbladder and to look for any sex differences in this parameter, since an increased gallbladder fossa size is a reported sign of liver fibrosis [6]. However, the primary aim of this study was to determine whether the volume of the gallbladder fossa would decrease with the absence of a gallbladder.

## Materials and methods

In this study, deidentified cadavers from the University of Texas Southwestern Medical Center (https://www.utsouthwestern.edu/research/programs/willed-body/) were utilized following permission and guidance from this program. Cadavers were embalmed with a mixture primarily containing ethyl alcohol, ethylene glycol, and phenol, with small amounts of formaldehyde and glutaraldehyde. Livers with their associated gallbladder were removed from embalmed cadavers that were used in a medical school anatomy course at Pacific Northwest University of Health Sciences (PNWU). All livers were included unless there was macroscopic evidence of liver disease or liver disease was listed as one of the causes of death for the individual. Thus, livers were studied from 34 cadavers (19 females, 15 males) with a mean age of 84.1 ± 10.8 years (range of 60–105 years).

**Fig 1:**
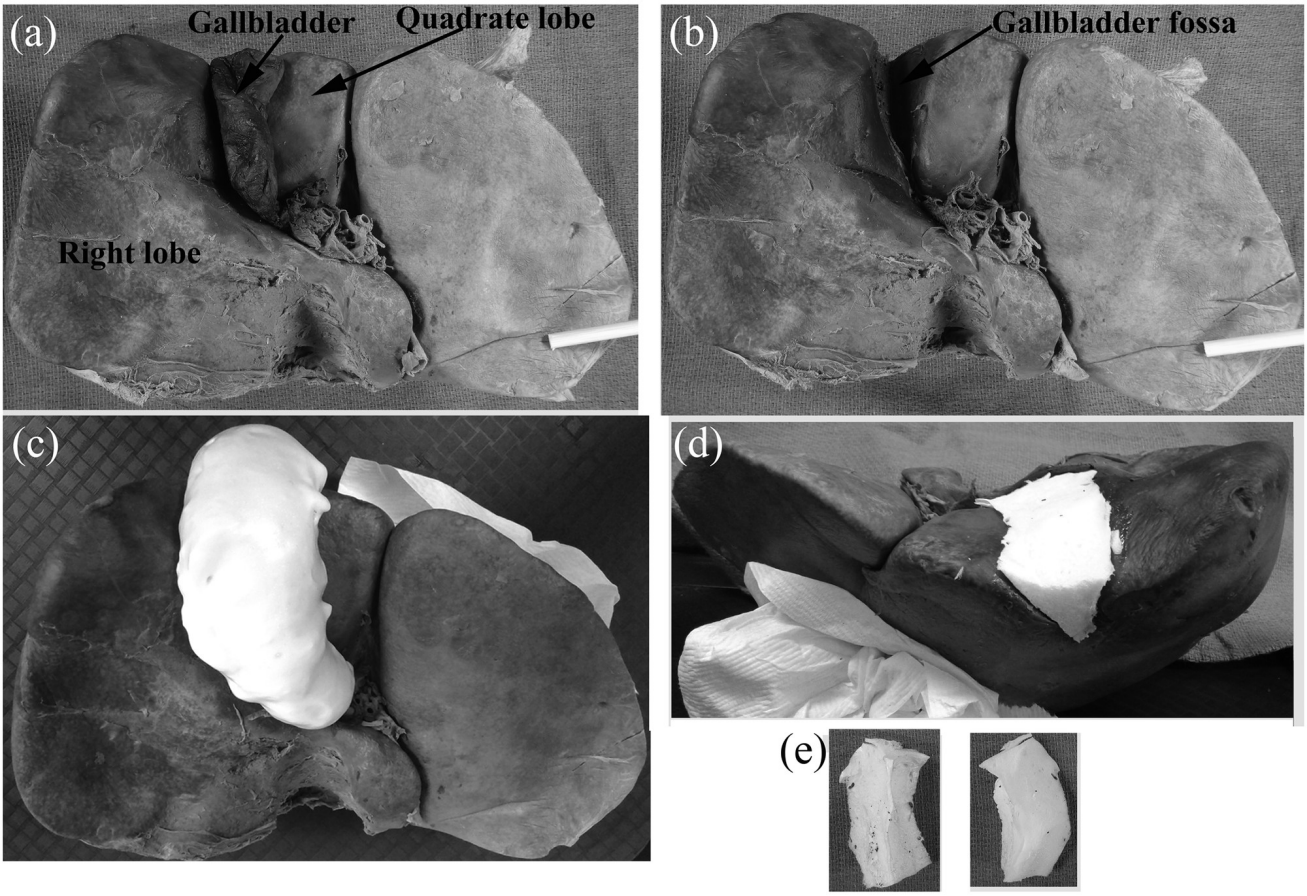
Evaluation of gallbladder fossa after liver removal. **(a)** Visceral surface of liver demonstrating a gallbladder located in its fossa between the right and quadrate lobes of the liver. **(b)** Same liver as is (a) with the gallbladder removed, demonstrating the gallbladder fossa. **(c)** Same liver with foam material added to the fossa and allowed to expand. **(d)** Same liver after foam material trimmed to lie flush with the visceral surface of the liver. **(e)** Two views of the mold in (d) after removal from the fossa.

Examination of the visceral surface of each liver identified eight cadavers without gallbladders (six females and two males, mean age of 83.9 ± 6.6 years), and 26 cadavers with gallbladders (13 females, 13 males, mean age of 84.2 ± 11.9 years). Livers were photographed and weighed (both before and after gallbladder removal if a gallbladder was present) (Fig 1a and 1b). The volume of the irregularly-shaped gallbladder fossa was determined by making a mold of the fossa and then calculating the volume of this mold from the weight of the mold and the density of the mold material. This mold was made by instilling an insulating foam sealant (Dow, Midland, MI, USA) into the area of each gallbladder fossa (Fig 1c). The fully expanded foam was trimmed flush with the surrounding liver parenchyma (Fig 1d) and then removed from the fossa (Fig 1e) and allowed to air-dry before being weighted on an analytical balance (Mettler Toledo MS105, Columbus, OH, USA).

The overall density of the dried foam material was determined using the pieces of excess foam material that had been trimmed from the fossa mold. After weighing these pieces of foam on an analytical balance, their volume was determined by displacement of water in a partially-filled graduated cylinder, and the density of the foam was calculated. The volume of each gallbladder fossa mold was determined by dividing the weight of the mold by the mean density of the foam material. Additionally, the dimensions of each irregularly-shaped fossa were estimated using a digital caliper (VWR, Radnor, PA, USA) to measure the largest distance for the depth, length, and width of each fossa mold.

The length of the femur was used to normalize the gallbladder fossa measurements to the overall size of the individual. For each cadaver, the length of the femur was determined by measuring from the most superior aspect of the femoral head to the most inferior aspect of the condylar surface. All individual data for this study are listed in the Supporting information tables. All weights were measured once by DR; femur lengths were measured once by RW; and depth/length/width measurements of the fossa molds were measured by both DR and RW, each measuring these twice, independently, at separate times. This study was classified as “non-human subjects” research since no information about living humans was obtained. Thus, IRB approval was not needed.

Statistical analyses were performed using the R environment (https://www.r-project.org/). The Shapiro-Wilk normality test was performed on each data set. If both parameters, whose means were to be compared, were normally distributed, Welch’s t-test was used to compare group means. However, if at least one of the parameters was not normally distributed, the nonparametric Mann Whitney U test was utilized. For the fossa depth/length/width measurements, intra-observer (ICC (3,1)) and inter-observer (ICC (3,k)) errors were determined. The Pearson product-moment correlation coefficient was utilized for correlation analyses. A *P*-value of <0.05 was considered statistically significant.

## Results

The mean volume of the gallbladder fossae from livers with gallbladders (31.01 ± 17.82 ml) was significantly greater than fossae in livers without gallbladders (8.75 ± 4.72 ml) (*P*<0.0001) (Table 1). The fossae were significantly larger in livers with gallbladders, even when corrected for the overall mass of the liver and size of the individual, as determined from the length of the femur (Table 1). Additionally, all three linear measurements taken of the fossae molds (depth, length, and width) were significantly larger in the livers with a gallbladder (*P*<0.05). Analysis of the intra-observer (ICC (3,1)) and inter-observer (ICC (3,k)) errors on these linear measurements, measured twice by two different individuals, demonstrated excellent reliability with ICC values of 0.94 and above (S1–S3 Tables). The largest percentage difference in the three linear measurements between those with and without gallbladders was in the mean depth of the fossa (Table 1).

**Table 1:** Comparison of gallbladder fossa parameters in livers from cadavers with and without gallbladders.

Gallbladder Fossa Parameter	With Gallbladder	No Gallbladder	Test of Significance	% Difference between means
	Mean ± SD	Mean ± SD		
	(Median)	(Median)	*P*-value	
	(Range)	(Range)	(Mann-Whitney = *MW* or Welch’s t-test = *W*)	
	Normality test P-value	Normality test P-value		
**Volume (ml)**	**31.01 ± 17.82**	**8.75 ± 4.72**	***P*<0.0001**	**71.8%**
	(28.64)	(8.03)	*(MW*, *z = -4*.*33)*	
	(7.20–91.77)	(3.19–16.57)		
	*P* = 0.0022	*P* = 0.4575		
**Volume (ml)/Liver weight (kg)**	**30.60 ± 19.57**	**10.13 ± 6.30**	***P* = 0.0002**	**66.9%**
	(25.00)	(8.51)	*(MW*, *z = -3*.*74)*	
	(8.59–86.46)	(3.21–20.59)		
	*P* = 0.0005	*P* = 0.2872		
**Volume (ml)/Femur length (mm)**	**0.068 ± 0.038**	**0.020 ± 0.01**	***P*<0.0001**	**70.8%**
	(0.063)	(0.017)	*(MW*, *z = -4*.*41)*	
	(0.019–0.186)	(0.007–0.039)		
	*P* = 0.0019	*P* = 0.6378		
**Depth (mm)**	**21.34 ± 6.17**	**8.02 ± 2.72**	***P*<0.0001**	**63.3%**
	(21.48)	(7.85)	*(W*, *t = -8*.*67)*	
	(9.98–36.97)	(4.85–12.52)		
	*P* = 0.8326	*P* = 0.4980		
**Length (mm)**	**65.04 ± 12.76**	**51.02 ± 8.66**	***P = 0*.*0024***	**21.6%**
	(64.08)	(49.02)	*(W*, *t = -3*.*54)*	
	(34.89–92.72)	(37.17–65.98)		
	*P* = 0.9894	*P* = 0.8495		
**Width (mm)**	**51.39 ± 11.97**	**36.19 ± 14.20**	***P* = 0.0119**	**29.6%**
	(49.84)	(31.75)	*(MW*, *z = -2*.*52)*	
	(33.30–79.24)	(20.89–60.79)		
	*P* = 0.0379	*P* = 0.2244		

**Fig 2:**
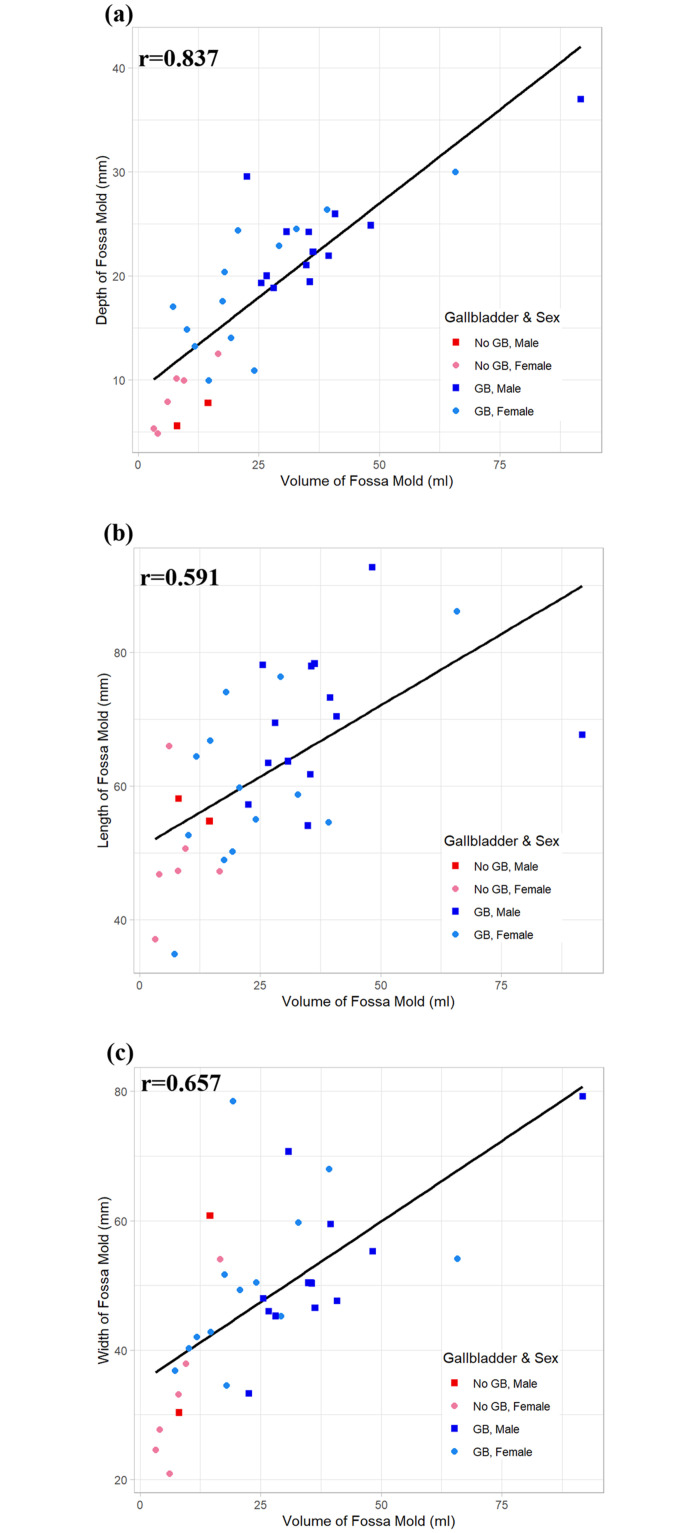
Plots gallbladder fossae volumes versus different dimensions of the fossa mold for livers with (blue) and without (red) gallbladders (males (squares), females (circles)), showing Pearson’s correlation coefficient (r) for each comparison. **(a)** Fossa volume versus fossa depth. **(b)** Fossa volume versus fossa length. **(c)** Fossa volume versus fossa width.

The correlation between the volume of the gallbladder fossae and its three dimensions (depth, length, and width) all were positive and significant (*P*< 0.05), with the strongest correlation being between the volume and depth of the fossae (Fig 2).

The finding that the volume of the gallbladder fossa was smaller in livers without gallbladders, suggested that the liver parenchyma expanded into that area, partially filling in the fossa. To evaluate whether this was due to overall liver hypertrophy or to a more local reaction, the liver weights were compared between the two groups. As seen in Table 2, there was no apparent difference in the liver weights between individuals with or without gallbladders, even when compensating for the difference in overall size of an individual by considering the length of their femur. Since most of the livers without gallbladders were from females, this analysis was repeated comparing only female livers with and without gallbladder; in this case, the liver weights were even more similar between livers with or without gallbladders (Table 2).

**Table 2:** Comparison of liver weights (raw and corrected for femur length) in cadavers with and without gallbladders.

Parameter	With Gallbladder	No Gallbladder	Test of Significance
	Mean ± SD	Mean ± SD	
	(Median)	(Median)	*P*-value
	(Range)	(Range)	(Mann-Whitney = *MW* or Welch’s t-test = *W*)
	Normality test *P*-value	Normality test *P*-value	
**Liver weight (g) (♂**and **♀)**	**1076.4 ± 332.9**	**914.4 ± 195.6**	***P* = 0.191**
	(1031.5)	(867.0)	*(MW*, *z = -1*.*31)*
	(648.1–2013.1)	(705.1–1349.9)	
	*P* = 0.0053	*P* = 0.0592	
**Liver weight (g)/Femur length (mm) (♂** and **♀)**	**2.38 ± 0.69**	**2.08 ± 0.39**	***P* = 0.2204**
	(2.20)	(2.13)	*(MW*, *z = -1*.*23)*
	(1.57–4.50)	(1.46–2.77)	
	*P* = 0.0002	*P* = 0.9094	
**Liver weight (g) (♀ only)**	**899.4 ± 140.5**	**876.5 ± 71.9**	***P* = 0.5911**
	(918.0)	(867.0)	*(W*, *t = -0*.*538)*
	(648.1–1138.9)	(790.2–993.0)	
	*P* = 0.9981	*P* = 0.8736	
**Liver weight (g)/Femur length (mm) (♀ only)**	**2.11 ± 0.28**	**2.08 ± 0.21**	***P* = 0.7961**
	(2.17)	(2.13)	*(W*, *t = -0*.*264)*
	(1.57–2.64)	(1.80–2.36)	
	*P* = 0.9890	*P* = 0.3553	

In addition to studying the effect of cholecystectomy, the volume of the gallbladder fossae were compared between males (n = 13) and females (n = 13) for the 26 livers with gallbladders. There was a wide range of fossa volumes in both males and females. While the overall fossa volume was significantly larger in male livers, this significance disappeared when fossa volume was corrected for the overall size of the liver or the size of the individual by dividing the fossa volume by the weight of the liver or the length of the femur respectively (Table 3).

**Table 3:** Gallbladder fossa parameters in livers with gallbladders, segregated by sex.

Parameter	Female	Male	Mann-Whitney
	Mean ± SD	Mean ± SD	*P*-value
	(Median)	(Median)	*z-score*
	(Range)	(Range)	
	Normality test *P*-value	Normality test *P*-value	
**Fossa volume (ml)**	**23.84 ± 15.56**	**38.18 ± 17.56**	***P* = 0.0051**
	(19.29)	(35.39)	*z = -2*.*80*
	(7.20–65.79)	(22.56–91.77)	
	*P* = 0.0228	*P* = 0.0005	
**Fossa volume (ml)/Liver weight (kg)**	**28.24 ± 21.29**	**32.96 ± 18.24**	***P* = 0.1534**
	(21.09)	(30.88)	*z = -1*.*43*
	(8.59–80.23)	(13.68–86.46)	
	*P* = 0.0091	*P* = 0.0026	
**Fossa volume (ml)/Femur length (mm)**	**0.056 ± 0.038**	**0.080 ± 0.035**	***P* = 0.0164**
	(0.044)	(0.073)	*z = -2*.*40*
	(0.019–0.160)	(0.047–0.186)	
	*P* = 0.0114	*P* = 0.0007	

Finally, since gallbladder fossa volume tended to be smaller in females than males normally, the effect of removing the gallbladder on fossa volume was evaluated using only the female samples. As seen in Table 4, even considering only female samples, the gallbladder fossa volume, depth, and width were significantly smaller in livers without a gallbladder than in livers with a gallbladder.

**Table 4:** Comparison of gallbladder fossa parameters in livers from female cadavers with and without gallbladders.

Gallbladder Fossa Parameter	With Gallbladder	No Gallbladder	Test of Significance	% Difference between means
	Mean ± SD	Mean ± SD		
	(Median)	(Median)	*P*-value	
	(Range)	(Range)	(Mann-Whitney = *MW* or Welch’s t-test = *W*)	
	Normality test P-value	Normality test P-value		
**Volume (ml) (♀ only)**	**23.84 ± 15.56**	**7.90 ± 4.86**	***P* = 0.0022**	**66.8%**
	(19.29)	(7.07)	*(MW*, *z = -3*.*06)*	
	(7.20–65.79)	(3.19–16.57)		
	*P* = 0.0228	*P* = 0.3684		
**Depth (mm) (♀ only)**	**18.93 ± 6.32**	**8.46 ± 2.99**	***P* = 0.0001**	**55.3%**
	(17.59)	(8.94)	*(W*, *t = -4*.*91)*	
	(9.98–29.97)	(4.85–12.52)		
	*P* = 0.7115	*P* = 0.6203		
**Length (mm) (♀ only)**	**60.22 ± 13.45**	**49.21 ± 9.39**	***P* = 0.0590**	**18.3%**
	(58.77)	(47.32)	*(W*, *t = -2*.*06)*	
	(34.89–86.16)	(37.17–65.98)		
	*P* = 0.9894	*P* = 0.2333		
**Width (mm) (♀ only)**	**50.28 ± 12.56**	**33.07 ± 11.95**	***P* = 0.0162**	**34.2%**
	(49.35)	(30.43)	*(W*, *t = -2*.*87)*	
	(34.51–78.49)	(20.89–54.10)		
	*P* = 0.3258	*P* = 0.4709		

## Discussion

In this study, it was assumed that cadavers without gallbladders had undergone previous cholecystectomies. While the absence of a gallbladder in a cadaver with no accompanying medical history does not negate the possibility of a congenitally absent organ, gallbladder atresia is very rare [7]. Conversely, gallbladder disease and subsequent removal of the gallbladder is very common, with an estimated 700,000 cholecystectomies being performed annually in the United States [8] and Japanese surgeons agreeing that cholecystectomy by laparoscopic approaches is recommended for acute cholecystitis in patients at their first hospital admission [9], if the patient and surgical setting meet other criteria [10]. Gallbladder disease occurs with an increasing frequency with aging and is more common in women then in men, with approximately 50% of females and 16% of males over 70 years of age reported as having gallstones [11]. Thus, it is not surprising that in this cadaver population with a mean age of 84 years, about 24% of the livers had no gallbladder present, and that the majority of livers without gallbladders were from female cadavers.

The main finding in this cadaveric study was that the volume of the gallbladder fossa was significantly less in livers from individuals without gallbladders compared to those with gallbladders, even when correcting for the overall size of the liver and the body size. The greatest relative change in the dimensions of the fossa occurred in the depth of this structure, although all three dimensions (depth, length, and width) were significantly decreased in the absence of a gallbladder, and there was a positive correlation between all three of these dimensions and the overall volume of the fossa. Knowledge that the gallbladder fossa is significantly smaller when a gallbladder is absent could be important when using fossa size as an early indicator for liver fibrosis [6]. Additionally, this study provided some preliminary data on the range of the volume of gallbladder fossae in males and females with gallbladders.

An unanswered question that these findings pose is the mechanism of this reduction in the fossa volume. The liver is well known for its regenerative capabilities. When liver tissue is removed, regeneration occurs, keeping the liver mass unchanged relative to body mass, a phenomenon termed “hepatostat” [3, 12]. Recent studies have demonstrated that liver progenitor cells lie in a transition zone between the hepatocytes and the biliary tree, and that liver regeneration associated with the bile canaliculi involves the actin cytoskeleton and the Hippo-YAP pathway, stimulated by bile acid overload [12, 13]. Since individuals undergoing a cholecystectomy may have had abnormalities that influenced the bile excretory pathway, the liver responses described in these studies presumably would have involved the entire liver; yet overall, in the present study, there was no apparent generalized liver hypertrophy after gallbladder removal, since weights were not different in livers with or without gallbladders.

Rather than eliciting a reaction from the entire liver, a much more localized response in the area surrounding the gallbladder fossa could be occurring in response to cholecystectomy. Liver parenchymal reaction in the area surrounding a diseased gallbladder can be detected with various imaging techniques, both before and after cholecystectomy [14–16]. While the partial “filling in” of an empty gallbladder fossa could be the result of a localized inflammatory response due to the traumatic nature of the gallbladder disease and subsequent surgery, a more likely explanation is that this area of the liver is responding, in the longer term, to new dynamics related to a change in the localized mechanostimulatory inputs after removal of the gallbladder. Passive extracellular features such as the topography and stiffness of surrounding areas can influence cellular responses [17]. There is precedence for a change in liver size and shape after adjacent organs, such as the spleen, are removed surgically [18]. Additionally, the speculation that the shape of the liver can fit in to the space available is suggested by the wide range of “normal” liver shapes that have been documented [19, 20].

While the present study highlights some novel findings, it also has several limitations. The gallbladder fossae were observed only at a single time point, not before and then after cholecystectomy. Without a medical history for the cadavers, it is unknown how long before death the gallbladders were removed, and thus how rapidly the fossa dimensions actually changed. However, regeneration of liver volume posttransplant in humans is rapid, with significant changes occurring in the first two weeks and maximal organ volume being reached by two months [21]. Another limitation is the small sample size, particularly in the number of male livers without gallbladders, as well as the fact that there were large differences in the overall sample size between livers with gallbladders and those without gallbladders. Thus, conducting future studies to increase the sample size, particularly of livers without gallbladders would be desirable. Additionally, histopathology was not performed on the livers; yet, all livers were handled similarly and if the liver was grossly abnormal, it was not included in this study.

In conclusion, the indentation on the visceral surface of the liver that normally houses the gallbladder was examined in cadaver livers. In livers with a gallbladder present, the range of this fossa’s volume was large. Before correcting for overall liver weight and the size of the individual, the average volume of the gallbladder fossa was greater in males than in females, but this difference disappeared after the fossa volume was corrected for overall liver weight and the size of the individual. The most noteworthy finding in the present study was that the fossa volume was significantly decreased in livers lacking gallbladders, even after correcting for the liver weight and size of the individual. While this finding provides useful metrics when accessing gallbladder fossa size in instances of liver disease, what is more intriguing are the questions it raises on the mechanisms involved in this localized liver hypertrophy. The answers to these questions await further research.

## Supporting information

S1 Table Reliability of fossa linear measurements—Depth. (PDF)

S2 Table Reliability of fossa linear measurements—Length. (PDF)

S3 Table Reliability of fossa linear measurements—Width. (PDF)

S4 Table Livers WITHOUT gallbladders. (PDF)

S5 Table Livers WITH gallbladders. (PDF)
