# Supplementary material for: Gallbladder fossa volume decreased in livers without gallbladders: A cadaveric study
Source: PLoS One. 2021 Sep 23;16(9):e0257848. doi: 10.1371/journal.pone.0257848 (PMC8459945; doi:10.1371/journal.pone.0257848)
Supplement: S1 Table — (PDF) [file pone.0257848.s001.pdf]

S1 Table

Reliability of fossa linear measurements - Depth

cadaver number	Depth of fossa mold (mm) DR1	Depth of fossa mold (mm) DR2	Depth of fossa mold (mm) RW1	Depth of fossa mold (mm) RW2	Mean Depth of fossa mold (mm)
1	20.31	20.61	20.94	22.25	21.03
2	10.15	10.79	9.81	9.90	10.16
3	32.31	28.80	27.86	29.15	29.53
4	28.96	29.36	30.68	30.87	29.97
5	7.87	8.14	8.03	7.51	7.89
6	6.00	4.78	5.81	5.73	5.58
7	10.47	10.99	11.34	10.74	10.89
8	23.31	23.92	24.50	25.19	24.23
9	12.70	12.35	12.70	12.34	12.52
10	17.21	17.32	17.93	17.88	17.59
11	22.30	21.45	22.00	22.01	21.94
12	22.12	21.49	24.90	23.00	22.88
13	26.42	26.00	26.52	26.56	26.38
14	22.05	21.77	22.69	22.78	22.32
15	14.70	13.54	16.06	15.17	14.87
16	19.78	19.77	21.10	20.82	20.37
17	19.40	18.75	19.32	19.78	19.31
18	5.32	5.61	5.10	5.37	5.35
19	24.17	23.38	24.54	24.66	24.19
20	12.82	14.83	14.42	14.17	14.06
21	37.02	36.41	37.34	37.09	36.97
22	24.44	23.00	25.87	26.10	24.85
23	9.94	7.46	10.86	11.67	9.98
24	26.27	25.80	26.17	25.57	25.95
25	24.20	24.08	24.90	24.92	24.53
26	18.72	18.05	18.65	19.89	18.83
27	13.41	12.46	13.34	13.73	13.24
28	7.60	7.88	7.83	7.89	7.80
29	15.78	17.11	17.49	17.74	17.03
30	17.44	18.58	20.64	21.00	19.42
31	25.22	23.51	24.56	24.13	24.36
32	19.58	19.47	20.48	20.48	20.00
33	3.85	5.14	5.19	5.20	4.85
35	9.27	8.51	11.54	10.59	9.98

ICC (3,1) DR = 0.99 **ICC (3,1) RW = 0.99** **ICC (3,k) DR/RW = 0.99**
