# Supplementary material for: Gallbladder fossa volume decreased in livers without gallbladders: A cadaveric study
Source: PLoS One. 2021 Sep 23;16(9):e0257848. doi: 10.1371/journal.pone.0257848 (PMC8459945; doi:10.1371/journal.pone.0257848)
Supplement: S2 Table — (PDF) [file pone.0257848.s002.pdf]

S2 Table

Reliability of fossa linear measurements - Length

cadaver number	Length of fossa mold (mm) DR1	Length of fossa mold (mm) DR2	Length of fossa mold (mm) RW1	Length of fossa mold (mm) RW2	Mean Length of fossa mold (mm)
1	60.27	58.82	48.64	48.61	54.09
2	47.46	47.42	47.14	47.50	47.38
3	58.18	56.68	56.99	57.00	57.21
4	83.18	89.77	85.87	85.80	86.16
5	66.02	65.05	66.19	66.67	65.98
6	59.13	58.72	56.73	58.01	58.15
7	61.69	53.80	51.91	52.83	55.06
8	76.67	62.15	57.19	58.81	63.71
9	45.17	48.65	46.94	48.29	47.26
10	51.46	49.75	46.73	48.02	48.99
11	73.64	72.98	72.76	73.52	73.23
12	75.22	76.26	76.84	77.12	76.36
13	53.66	59.21	51.04	54.39	54.58
14	78.19	77.72	78.69	78.54	78.29
15	51.73	52.29	54.24	52.53	52.70
16	73.94	73.70	74.41	74.38	74.11
17	80.20	74.58	77.54	80.10	78.11
18	41.88	34.62	33.70	38.46	37.17
19	63.80	61.15	61.21	60.78	61.74
20	44.97	52.16	49.35	54.53	50.25
21	66.17	67.91	68.14	68.36	67.65
22	92.10	92.21	94.87	91.68	92.72
23	52.06	42.06	54.83	53.67	50.66
24	69.65	71.17	70.34	70.61	70.44
25	59.20	59.06	58.69	58.14	58.77
26	69.22	70.97	69.07	68.48	69.44
27	64.91	64.89	62.95	65.09	64.46
28	54.37	53.21	55.55	55.87	54.75
29	37.68	32.78	34.61	34.49	34.89
30	77.91	77.94	78.29	77.56	77.93
31	55.60	62.37	60.31	60.79	59.77
32	58.70	59.63	67.45	68.15	63.48
33	45.87	45.46	48.24	47.75	46.83
35	62.33	64.53	69.27	71.04	66.79

ICC (3,1) DR = 0.94 **ICC (3,1) RW = 0.99** **ICC (3,k) DR/RW = 0.96**
