# Supplementary material for: Gallbladder fossa volume decreased in livers without gallbladders: A cadaveric study
Source: PLoS One. 2021 Sep 23;16(9):e0257848. doi: 10.1371/journal.pone.0257848 (PMC8459945; doi:10.1371/journal.pone.0257848)
Supplement: S3 Table — (PDF) [file pone.0257848.s003.pdf]

S3 Table

Reliability of fossa linear measurements - Width

cadaver number	Width of fossa mold (mm) DR1	Width of fossa mold (mm) DR2	Width of fossa mold (mm) RW1	Width of fossa mold (mm) RW2	Mean Width of fossa mold (mm)
1	48.98	54.02	47.53	51.07	50.40
2	32.49	33.20	33.51	33.46	33.17
3	33.78	35.14	31.99	32.30	33.30
4	55.94	56.45	51.75	52.53	54.17
5	20.32	22.21	19.75	21.27	20.89
6	29.21	32.09	31.03	29.03	30.34
7	52.88	49.07	49.93	50.00	50.47
8	53.87	75.36	76.78	76.69	70.68
9	54.93	53.33	54.16	53.99	54.10
10	48.38	51.95	52.89	53.71	51.73
11	59.09	58.69	60.08	59.92	59.45
12	45.39	44.58	45.31	45.88	45.29
13	67.14	68.97	68.51	67.26	67.97
14	45.65	46.72	46.80	47.02	46.55
15	38.92	36.90	42.60	42.83	40.31
16	33.73	34.89	34.74	34.69	34.51
17	46.45	51.17	47.22	47.12	47.99
18	22.24	24.21	25.61	26.47	24.63
19	48.52	51.78	51.41	50	50.43
20	78.85	79.28	79.72	76.09	78.49
21	78.67	80.05	77.89	80.33	79.24
22	54.18	56.14	56.18	54.49	55.25
23	35.39	34.93	40.48	40.84	37.91
24	46.16	47.09	48.50	48.74	47.62
25	56.05	59.72	61.30	61.82	59.72
26	44.91	44.59	46.29	45.27	45.27
27	42.69	42.81	41.71	40.88	42.02
28	59.92	60.90	61.70	60.65	60.79
29	33.13	37.52	38.53	38.26	36.86
30	47.45	49.01	52.84	52.01	50.33
31	45.27	48.60	51.89	51.62	49.35
32	46.07	46.41	46.28	45.15	45.98
33	27.11	29.63	26.91	27.15	27.70
35	40.63	42.87	43.80	43.85	42.79

ICC(3,1) DR = 0.96 **ICC (3,1) RW = 0.99** **ICC (3,k) DR/RW = 0.99**
