# Supplementary material for: Gallbladder fossa volume decreased in livers without gallbladders: A cadaveric study
Source: PLoS One. 2021 Sep 23;16(9):e0257848. doi: 10.1371/journal.pone.0257848 (PMC8459945; doi:10.1371/journal.pone.0257848)
Supplement: S4 Table — (PDF) [file pone.0257848.s004.pdf]

S4 Table

Livers WITHOUT gallbladders

cadaver number	Age (yrs)	Sex	weight of fossa mold (g)	Calculated volume of fossa (ml)	Depth of fossa mold (mm)	Length of fossa mold (mm)	Width of fossa mold (mm)	Liver weight (g)	Femur length (mm)
2	89	F	0.203	7.99	10.16	47.38	33.17	790.2	438
5	84	F	0.156	6.14	7.89	65.98	20.89	889.4	407
6	84	M	0.205	8.07	5.58	58.15	30.34	1349.9	488
9	73	F	0.421	16.57	12.52	47.26	54.10	913.5	428
18	86	F	0.081	3.19	5.35	37.17	24.63	993	421
23	95	F	0.241	9.49	9.98	50.66	37.91	844.6	396
28	80	M	0.369	14.53	7.80	54.75	60.79	705.7	483
33	80	F	0.102	4.02	4.85	46.83	27.70	828.5	452

S2 Table

Livers WITH gallbladders

cadaver number	Age (yrs)	Sex	weight of fossa mold (g)	Calculated volume of fossa (ml)	Depth of fossa mold (mm)	Length of fossa mold (mm)	Width of fossa mold (mm)	Liver weight (g)	Femur length (mm)
1	80	M	0.886	34.88	19.73	47.49	49.79	752	435
3	72	M	0.573	22.56	28.65	56.23	30.7	1164.2	482
4	105	F	1.671	65.79	29.95	81.89	48.94	820	411
7	92	F	0.61	24.02	10.14	50.89	52.04	1138.9	431
8	92	M	0.783	30.83	18.84	61.31	34.83	1333	464
10	80	F	0.445	17.52	17.04	47.76	48.44	1082.5	479
11	75	M	1.004	39.53	21.62	68.62	53.07	2013.1	447
12	67	F	0.741	29.17	21.32	64.58	39.11	984.5	440
13	89	F	0.994	39.13	26.16	53.41	65.46	648.1	414
14	91	M	0.922	36.30	22.21	78.84	45.72	1072.7	497
15	93	F	0.256	10.08	14.42	52.52	41.23	1011	420
16	84	F	0.456	17.95	20.69	72.16	34.38	778.7	403
17	76	M	0.65	25.59	18.79	73.85	44.77	1871.2	445
19	90	M	0.899	35.39	23.18	62.47	48.8	1242.4	479
20	87	F	0.49	19.29	14.54	79.05	44.64	961.8	461
21	93	M	2.331	91.77	36.39	65.2	89.47	1061.4	493
22	71	M	1.226	48.27	24.54	87.97	55.03	1553.6	502
24	67	M	1.038	40.87	26.48	68.01	45.01	1323.4	470
25	60	F	0.832	32.76	23.85	55.29	49.74	729.5	402
26	90	M	0.714	28.11	18.9	69.24	45.05	731.6	466
27	88	F	0.299	11.77	12.71	44.03	37.09	918	420
29	104	F	0.183	7.20	16.32	33.58	27.43	838.6	386
30	74	M	0.905	35.63	19.4	74.96	50.11	1122.8	504
31	95	F	0.525	20.67	24.23	46.15	49.82	838.1	453
32	73	M	0.677	26.65	19.27	53.81	45.76	1051.9	483
35	100	F	0.371	14.61	8.66	65.97	30.47	942.6	424
