# Supplementary material for: Gallbladder fossa volume decreased in livers without gallbladders: A cadaveric study
Source: PLoS One. 2021 Sep 23;16(9):e0257848. doi: 10.1371/journal.pone.0257848 (PMC8459945; doi:10.1371/journal.pone.0257848)
Supplement: S5 Table — (PDF) [file pone.0257848.s005.pdf]

**S5 Table**

**Livers WITH gallbladders.**

| cadaver number | Age (yrs) | Sex | weight of fossa mold (g) | Calculated volume of fossa (ml) | Depth of fossa mold (mm) | Length of fossa mold (mm) | Width of fossa mold (mm) | Liver weight (g) | Femur length (mm) |
|---|---|---|---|---|---|---|---|---|---|
| 1 | 80 | M | 0.886 | 34.88 | 21.03 | 54.09 | 50.40 | 752 | 435 |
| 3 | 72 | M | 0.573 | 22.56 | 29.53 | 57.21 | 33.30 | 1164.2 | 482 |
| 4 | 105 | F | 1.671 | 65.79 | 29.97 | 86.16 | 54.17 | 820 | 411 |
| 7 | 92 | F | 0.61 | 24.02 | 10.89 | 55.06 | 50.47 | 1138.9 | 431 |
| 8 | 92 | M | 0.783 | 30.83 | 24.23 | 63.71 | 70.68 | 1333 | 464 |
| 10 | 80 | F | 0.445 | 17.52 | 17.59 | 48.99 | 51.73 | 1082.5 | 479 |
| 11 | 75 | M | 1.004 | 39.53 | 21.94 | 73.23 | 59.45 | 2013.1 | 447 |
| 12 | 67 | F | 0.741 | 29.17 | 22.88 | 76.36 | 45.29 | 984.5 | 440 |
| 13 | 89 | F | 0.994 | 39.13 | 26.38 | 54.58 | 67.97 | 648.1 | 414 |
| 14 | 91 | M | 0.922 | 36.30 | 22.32 | 78.29 | 46.55 | 1072.7 | 497 |
| 15 | 93 | F | 0.256 | 10.08 | 14.87 | 52.70 | 40.31 | 1011 | 420 |
| 16 | 84 | F | 0.456 | 17.95 | 20.37 | 74.11 | 34.51 | 778.7 | 403 |
| 17 | 76 | M | 0.65 | 25.59 | 19.31 | 78.11 | 47.99 | 1871.2 | 445 |
| 19 | 90 | M | 0.899 | 35.39 | 24.19 | 61.74 | 50.43 | 1242.4 | 479 |
| 20 | 87 | F | 0.49 | 19.29 | 14.06 | 50.25 | 78.49 | 961.8 | 461 |
| 21 | 93 | M | 2.331 | 91.77 | 36.97 | 67.65 | 79.24 | 1061.4 | 493 |
| 22 | 71 | M | 1.226 | 48.27 | 24.85 | 92.72 | 55.25 | 1553.6 | 502 |
| 24 | 67 | M | 1.038 | 40.87 | 25.95 | 70.44 | 47.62 | 1323.4 | 470 |
| 25 | 60 | F | 0.832 | 32.76 | 24.53 | 58.77 | 59.72 | 729.5 | 402 |
| 26 | 90 | M | 0.714 | 28.11 | 18.83 | 69.44 | 45.27 | 731.6 | 466 |
| 27 | 88 | F | 0.299 | 11.77 | 13.24 | 64.46 | 42.02 | 918 | 420 |
| 29 | 104 | F | 0.183 | 7.20 | 17.03 | 34.89 | 36.86 | 838.6 | 386 |
| 30 | 74 | M | 0.905 | 35.63 | 19.42 | 77.93 | 50.33 | 1122.8 | 504 |
| 31 | 95 | F | 0.525 | 20.67 | 24.36 | 59.77 | 49.35 | 838.1 | 453 |
| 32 | 73 | M | 0.677 | 26.65 | 20.00 | 63.48 | 45.98 | 1051.9 | 483 |
| 35 | 100 | F | 0.371 | 14.61 | 9.98 | 66.79 | 42.79 | 942.6 | 424 |
